# Specific Increase in Joint Neutrophil Extracellular Traps and Its Relation to Interleukin 6 in Autoimmune Arthritis

**DOI:** 10.3390/ijms22147633

**Published:** 2021-07-16

**Authors:** Ayako Ohyama, Atsumu Osada, Hoshimi Kawaguchi, Izumi Kurata, Taihei Nishiyama, Tamaki Iwai, Akihito Ishigami, Yuya Kondo, Hiroto Tsuboi, Takayuki Sumida, Isao Matsumoto

**Affiliations:** 1 Division of Rheumatology, Department of Internal Medicine, Faculty of Medicine, University of Tsukuba, Tsukuba, Ibaraki 305-8575, Japan; a.ikuta1212@gmail.com (A.O.); atsumu-osada-8074@hotmail.co.jp (A.O.); s0311596@yahoo.co.jp (H.K.); izzybreezy.23@gmail.com (I.K.); taihei.nishiyama.5320710@gmail.com (T.N.); s2121354@s.tsukuba.ac.jp (T.I.); YuKond@md.tsukuba.ac.jp (Y.K.); Hiroto-Tsuboi@md.tsukuba.ac.jp (H.T.); tsumida@md.tsukuba.ac.jp (T.S.); 2 Ichihara Hospital, Tsukuba, Ibaraki 300-3295, Japan; 3 Molecular Regulation of Aging, Tokyo Metropolitan Institute of Gerontology, Itabashi-ku, Tokyo 173-0015, Japan; ishigami@tmig.or.jp

**Keywords:** neutrophil extracellular traps, interleukin-6, citrullinated proteins, rheumatoid arthritis

## Abstract

Neutrophils and their extracellular traps have been shown to play an important role in the pathogenesis of rheumatoid arthritis (RA), but the detailed mechanisms in joints are still unclear, and their regulation remains to be solved. Here, we explored neutrophil extracellular trap (NET)osis in experimental models of arthritis and further investigated the effects of interleukin-6 (IL-6) inhibition in neutrophils and NETosis. In skins of peptide GPI-induced arthritis (pGIA), citrullinated protein was detected as well as citrullinated histone expression in immunized skin but this was not specific to pGIA. Citrullinated histone expression in pGIA joints was specific to pGIA and was merged with neutrophil elastase, suggesting NETosis. Neutrophils in joints tend to upregulate IL-6 receptors when compared with bone marrow neutrophils. Administration of mouse anti-IL-6 receptor antibodies in pGIA suppressed arthritis in association with a decrease in neutrophil infiltration and NETosis in joints. In the plasma of RA patients, citrullinated protein was significantly reduced after tocilizumab treatment. Our results suggest that IL-6 enhances neutrophil chemotaxis and NETosis in inflammatory joints and could be the source of citrullinated proteins.

## 1. Introduction

Rheumatoid arthritis (RA) is a chronic autoimmune disease affecting approximately 1% of the population. It is characterized by multiple inflammation and destruction of the synovial joints.

Neutrophils are the most abundant cells in the inflamed joints of early-phase RA, causing joint damage and inducing the production of proinflammatory cytokines [1].

The recent discovery of a new process of neutrophil cell death called neutrophil extracellular trap (NET)osis has shed light on the pathogenesis of RA [2]. NETosis is essentially a mechanism to protect against infection via the release of NETs [3]. NETs are reticulate structures consisting of nuclear chromatin, peptidylarginine deminase (PAD), ROS, and other intracellular substances. PAD is an enzyme that converts arginine to citrulline, and during the process of NETosis, it causes histone citrullination and triggers chromatin decondensation, promoting the process of NETosis [4]. PAD also causes citrullination of other intracellular proteins, and when NETs are released from the cell, the citrullinated proteins are also released.

The production of anti-citrullinated protein antibodies (ACPAs) is known to precede the onset of RA and is thought to be a key factor in the pathogenesis of RA [5,6,7], and is clearly linked to the HLA-DR shared epitope [8]. Furthermore, PAD4 SNPs have been shown to be associated with the risk of developing RA [9,10,11] indicating that citrullination by PAD is also important for the development of RA. ACPAs are thought to exacerbate arthritis through activation of macrophages via immunocomplex formation and promotion of proinflammatory cytokine production [12,13,14]. Previous reports revealed that NETosis is the main source of citrullinated autoantigens [15,16]. Smoking is a known risk factor for the development of RA, and it has been reported that citrullinated proteins are also produced in lung tissue [17], and the level of ACPAs has been reported to correlate with the level of NETs in sputum [18,19]. Thus, NETosis, with the production of citrullinated proteins, which become the corresponding antigens of ACPAs, may be considered to be one of the important pathogenesis factors of RA.

Glucose-6-phosphate isomerase (GPI) is a ubiquitous glycolytic enzyme, and was identified as a pathogenic autoantigen in K/BxN arthritic mice [20]. Immunization with recombinant human GPI could induce arthritis in DBA/1 mice (GPI-induced arthritis; GIA) [21]. CD4+ T cells play a critical role in GPI-induced arthritis, and the effect of biological agents including anti-interleukin-6 (IL-6) receptor antibodies is similar to that found in RA patients [22,23]. Immunization of a specific peptide that is a major CD4+ T cell epitope of GPI could induce arthritis in DBA/1 mice (peptide GPI-induced arthritis; pGIA) [24,25].

We previously reported a specific increase in a citrullinated protein of 120 kDa, citrullinated inter-alpha-trypsin-inhibitor heavy chain 4 (ITIH4), in the blood and joints of pGIA mice and of RA patients. The blood levels of citrullinated ITIH4 fluctuated according to the arthritis scores of the pGIA mice and the disease activity of the RA patients, whilst antibodies to the cit-ITIH4 epitope were specifically observed in the RA patients [26]. In addition, citrullinated protein was also detected in the joints of pGIA, and peptidyl arginine deiminase (PAD) inhibition decreased the citrullinated protein in the joints and suppressed arthritis [27]. However, the pathogenesis of pGIA and the source of the citrullinated protein remain unclear.

Here, we investigated the presence of citrullinated protein in the prearthritic phase of pGIA and also investigated the generation of NETosis in the skin and joints of pGIA. We found citrullinated protein production in immunized skin in the prearthritic phase and NETosis in the skin and joints of pGIA. In addition, treatment with anti-IL-6 receptor antibodies suppressed the arthritis via reduction in neutrophil infiltration and NETosis, resulting in decreased amounts of plasma citrullinated proteins.

## 2. Results

### 2.1. Citrullinated Protein Expression in Immunized Skin of pGIA

**Figure 1:**
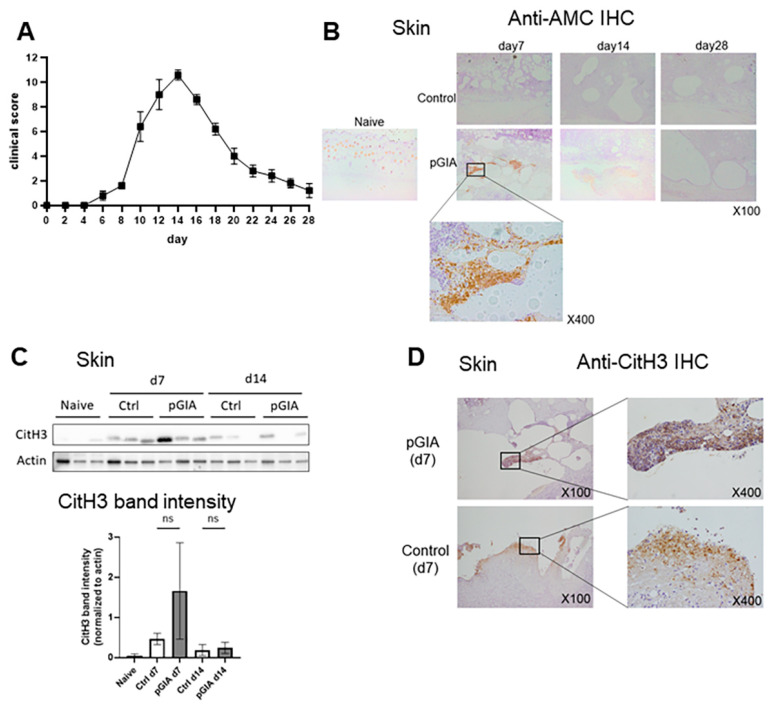
Citrullinated protein expression in the immunized skin of pGIA mice. (**A**) The clinical score of pGIA (means ± SEM), n = 5. (**B**) Skin sections were immunohistochemically stained with anti-modified citrulline (AMC) antibodies in pGIA or control mice. Hematoxylin was used as the counterstain. (**C**) Skin lysates from the pGIA mice and the control mice at days 0, 7, and 14 were subjected to Western blot analysis using anti-citrullinated histone 3 (CitH3) antibodies. Three mice were examined, and *p* values were calculated using ordinary one-way ANOVA with the post hoc Tukey test for multiple comparisons; (**D**) Skins were immunohistochemically stained with anti-CitH3 antibodies in the pGIA and control mice. Hematoxylin was used as the counterstain.

DBA/1 mice were immunized by indermal injection at the base of the tails with either peptide GPI325-339 (pGPI) in complete Freund’s adjuvant (CFA) or CFA only (as the vehicle control). Arthritis appeared at day 7–8, reached its peak severity at day 14, and then gradually resolved (Figure 1A).

We previously reported that intra-articular citrullinated protein expression was observed on day 14 and disappeared after administration of a PAD inhibitor in pGIA [27]. In this study, we focused on citrullinated protein expression in other organs. We first analyzed immunized skin, where citrullinated proteins are known to be abundantly expressed [28]. Immunohistochemistry (IHC) using anti-modified citrullinated protein (AMC) antibodies revealed citrullinated protein expression in the skin of the pGIA mice at days 7 and 14 but not in the skin of the control mice (Figure 1B). The citrullinated protein was surrounded by inflammatory cells, mainly polymorphonuclear cells, infiltrating the subcutaneous tissue in the injection areas.

As the citrullinated protein surrounded by neutrophils in the dermal tissue preceded that observed in the articular tissue, we speculated that the production of citrullinated proteins in the skin via NETosis could trigger the induction of arthritis.

Citrullinated histones are one of the citrullinated proteins released in the process of NETosis; therefore, their presence suggests NETosis. We assessed citrullinated histone 3 (CitH3) expression in immunized skin by Western blot (WB) analysis and IHC. Although CitH3 was detected in the pGIA skin on days 7 and 14, it was also detected in the vehicle control skin, i.e., it is not specific to pGIA (Figure 1C,D).

These findings suggested an unexpected presence of citrullinated proteins in the skin as a result of immunization, but NETosis in the skin is not a specific trigger for arthritis.

### 2.2. Citrullinated Histone 3 Expression in the Arthritic Joints of pGIA

We next explored the expression of CitH3 in the joints of pGIA, where we previously found the expression of citrullinated protein [26,27].

**Figure 2:**
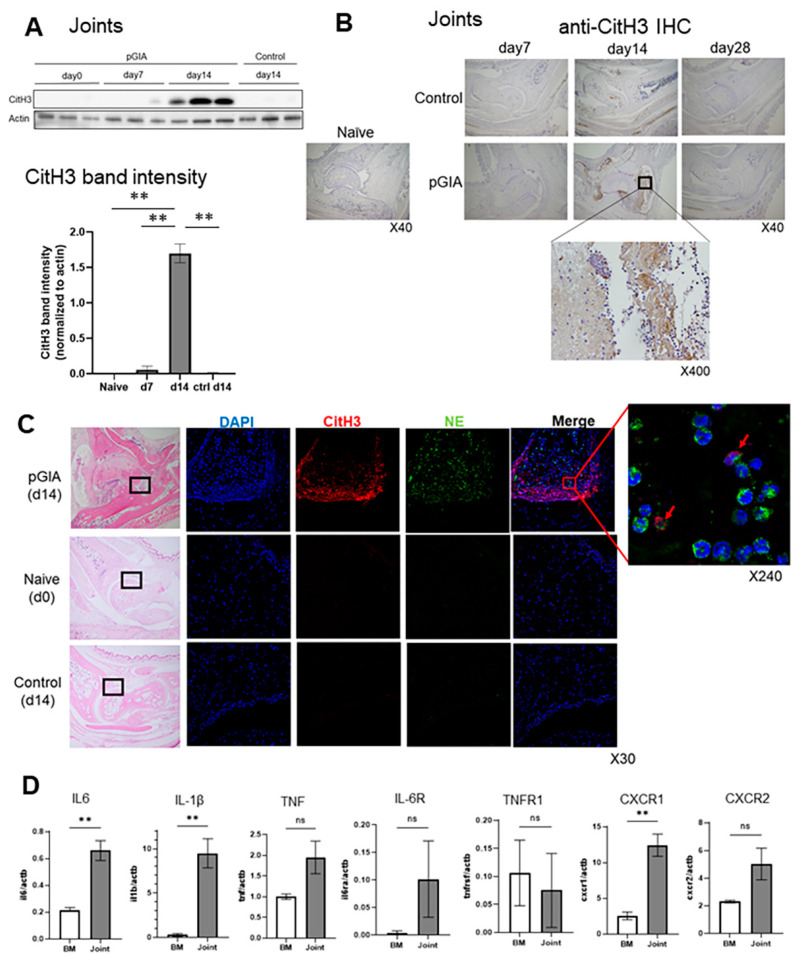
Citrullinated histone 3 (CitH3) expression in the arthritic joints of pGIA mice. (**A**) Joint lysates from pGIA mice at days 0, 7, and 14 and from control mice at day 14 were subjected to Western blot analysis using anti-CitH3 antibodies. Three mice were examined, and *p* values were calculated using ordinary one-way ANOVA with the post hoc Tukey test for multiple comparisons; ** *p* < 0.01. (**B**) Joint sections from pGIA and control mice were immunohistochemically stained with anti-CitH3 antibodies. Hematoxylin was used as the counterstain. (**C**) Immunofluorescence using anti-CitH3 antibodies and anti-neutrophil elastase (NE) antibodies was performed on joint sections from the pGIA mice at day 14 and from the naïve mice and control mice at day 14. DAPI was used for DNA staining. Box was enlarged image of the located portion, and red arrows are overlayed portion with CitH3 and NE. (**D**) Reverse transcription quantitative PCR was performed with neutrophils from the joints and bone marrow of the pGIA mice at day 14. Each gene expression value was normalized to actin. Data indicate means ± SEMs. *p* values were calculated using the unpaired *t*-test; ** *p* < 0.01.

WB of pGIA joint samples identified significant CitH3 overexpression in the arthritic joints of pGIA (Figure 2A). Then, we performed IHC to assess the distribution of CitH3 protein in joints and identified CitH3 in the synovium of inflamed joints of pGIA, surrounded by an abundant infiltrate of polymorphonuclear cells. CitH3 was highly expressed on day 14, with exacerbation of arthritis, and disappeared on day 28 (Figure 2B). Immunofluorescence (IF) of CitH3 and neutrophil elastase (NE) detected their expression in close proximity in the pGIA joints, and some of them were merged (Figure 2C), suggesting that NETosis occurs in the extreme phase of arthritis.

Then, we focused on neutrophils and compared the mRNA expression of cytokines, chemokines, and their receptors in localized neutrophils in joints with that in naïve neutrophils in bone marrow at day 14.

RT-qPCR revealed that IL-1β, IL-6, and CXCR1 in neutrophils in joints were relatively overexpressed (Figure 2D). IL-6 receptor, TNF, and CXCR2 also tended to be upregulated in the joints but not in a statistically significant manner.

### 2.3. Suppression of Arthritis by Treatment with MR16-1 in pGIA

As we mentioned before, biological agents, including anti-IL-6 receptor antibodies, had similar effects in GIA to those in RA [22,23], but the effects in pGIA had never been investigated. Additionally, it has been reported that IL-6 induces increased PAD4 expression [29] in neutrophils and that IL-6 induces NETosis [30]. However, the relationship between IL-6 and neutrophils in arthritis remains obscure. Considering these previous reports and IL-6 receptor expression in articular neutrophils, we hypothesized that IL-6 may affect neutrophil recruitment and functions in pGIA.

**Figure 3:**
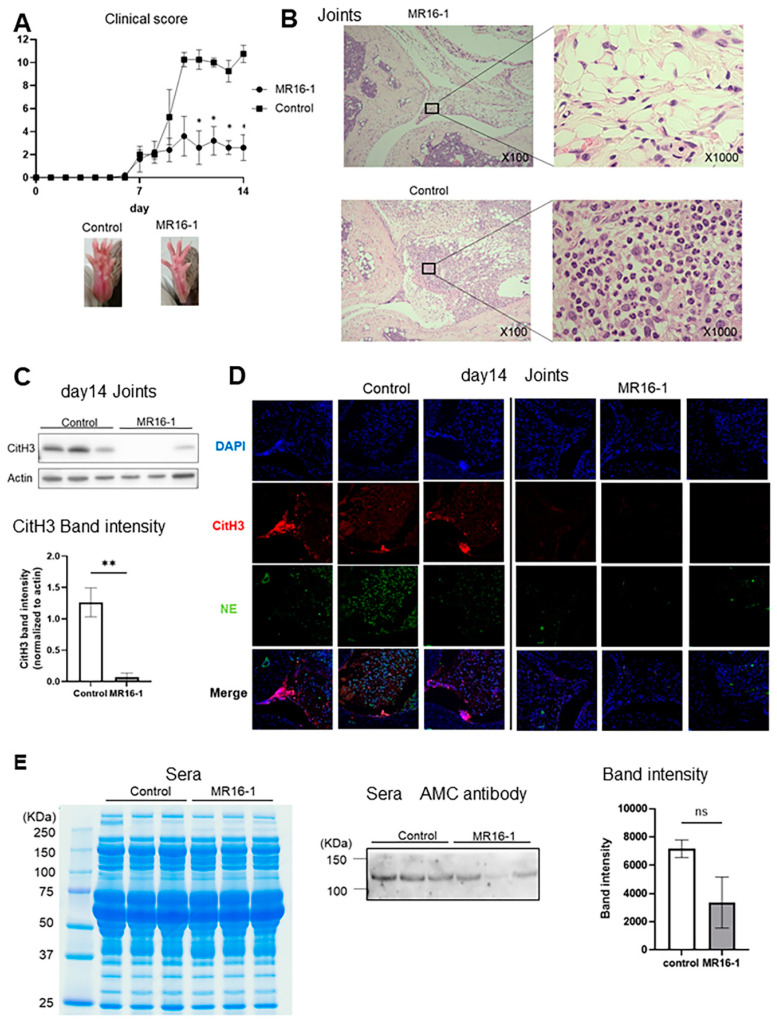
Suppression of arthritis obtained by treatment with MR16-1 in pGIA mice. (**A**) Clinical scores (means ± SEMs) of pGIA mice treated with MR16-1 (n = 5) or control IgG (n = 4), and representative images of ankle joints of pGIA and control mice. *p* values were calculated using two-way ANOVA with the post hoc Sidak multiple comparisons test. (**B**) Representative images of hematoxylin–eosin staining of pGIA joints treated with MR16-1 or control IgG. (**C**) Western blot analysis of the joint lysates of pGIA mice treated with MR16-1 or control IgG using anti-CitH3 antibodies. Three mice in each group were analyzed. Bars show means ± SEMs. *p* values were calculated using the unpaired *t*-test; ** *p* < 0.01. (**D**) Immunofluorescence using anti-CitH3 antibodies and anti-NE antibodies were performed on joint sections from the pGIA mice treated with MR16-1 and from the control mice (representative images from 3 mice in each group). DAPI was used for DNA staining. (**E**) Sera from pGIA mice treated with MR16-1 and control IgG were separated by SDS-PAGE then stained with Coomassie brilliant blue (CBB) or subjected to Western blot analysis using anti-modified citrulline antibodies. Three mice in each group were analyzed. *p* values were calculated using the unpaired *t*-test.

To investigate the relation between IL-6 and neutrophils in pGIA, we used MR16-1, which are mouse anti-IL-6 receptor antibodies. We immunized DBA1/J mice with pGPI and injected them intraperitoneally with 2 mg/body of MR16-1 or control IgG. The clinical score was significantly reduced in the MR16-1 treatment mice as compared with in the control mice (Figure 3A). Histological assessment of the ankle joint sections showed remarkable reduction in inflammatory cell infiltration and synovial hyperplasia by MR16-1 treatment (Figure 3B). Furthermore, WB of the joint samples at day 14 identified reduction in CitH3 expression in the MR16-1 treatment mice (Figure 3C), and IF revealed reduction in NE and CitH3 expression in the synovium of the treatment group (Figure 3D).

We previously reported a specific increase in a citrullinated protein of 120 kDa, the citrullinated form of ITIH4 (cit-ITIH4), in the blood of pGIA mice and RA patients, shown by mass spectrometry analysis. The blood levels of cit-ITIH4 fluctuated according to the arthritis scores of the pGIA mice and the disease activity of the RA patients [26]. We also assessed the citrullinated protein of 120 kDa in the MR16-1 treatment mice. The expression of citrullinated 120 kDa protein tended to be reduced in the MR16-1 treatment mice (Figure 3E). In addition, we recently made ITIH4-deficient mice, which clearly lost the 120 kDa bands in plasma in arthritic conditions (manuscript in preparation). This result supports that this 120 kDa band might be cit-ITIH4.

### 2.4. Reduction in 120 kDa Citrullinated Protein in RA Patients Treated with Tocilizumab

**Figure 4:**
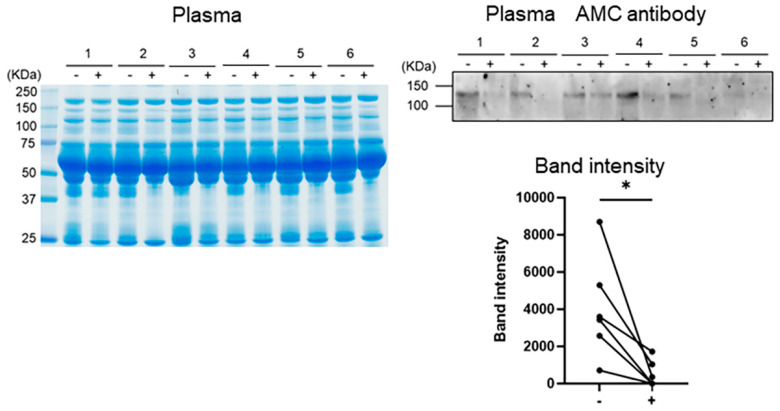
Reduction in citrullinated protein in RA patients treated with tocilizumab. Sera from RA patients at baseline or at 6 months after tocilizumab (TCZ) treatment were separated by SDS-PAGE then stained with CBB or were subjected to Western blot analysis with anti-modified citrulline (AMC) antibodies. Bars show means ± SEMs. *p* values were calculated using the unpaired *t*-test; * *p* < 0.05.

As a reduction in CitH3 as well as in the 120 kDa protein was observed in pGIA treated with MR16-1, we next assessed the changes in the 120 kDa protein in RA patients treated with tocilizumab (TCZ), humanized anti-IL-6 receptor antibodies. WB analysis with AMC antibodies revealed that the citrullinated protein of 120 kDa was significantly decreased after 6 months of treatment with TCZ (Figure 4).

## 3. Discussion

Here, we identified joint-specific induction of NETosis and that anti-IL-6 receptor antibodies suppressed the severity of arthritis via decreased neutrophilic infiltration and NETosis in the joints of pGIA.

Anti-citrullinated protein antibodies (ACPAs) are clearly linked to the HLA-DR shared epitope [8] and have been reported to be specific to RA and to precede the onset of the disease [5,6]. Previous reports revealed that NETosis is the main source of citrullinated autoantigens in RA [15,16]. If joint-specific citrullinated antigens have been upregulated, ACPAs made immunocomplexes locally, and possibly enhanced arthritis severity.

Initially, we speculated that NETosis in immunized skin might trigger the development of pGIA, since we found citrullinated protein expression in the skin of pGIA at day 7. However, the CitH3 expression in the skin was not specific to pGIA. A variety of nonsterile or sterile stimuli can cause NETosis, such as bacteria, fungi, crystals, ionomycin, and phorbol 12-myristate 13-acetate (PMA). In the immunized skin of both pGIA and control mice, chemical inflammation accompanied by abundant neutrophilic infiltration is observed. These nonspecific stimulations by emulsions may induce NETosis in the skin of both pGIA and control mice.

In contrast to in the skin, NETosis in the joints was specific to pGIA. CitH3 was most highly expressed on day 14, the extreme phase of arthritis, and disappeared on day 28 (Figure 1B). Although the cause of decreased presentation of CitH3 on day 28 has not been clarified, the pGIA was improved on day 28 (Figure 1A). As arthritis resolved, infiltration of neutrophils and synovial hyperplasia also disappeared, Thus, we assume that the stimulation, such as inflammatory cytokines and immune complexes that trigger NETs, may also have been reduced at day 28.

We previously found that citrullinated protein detected with AMC antibodies was specifically expressed in pGIA joints at day 14 and suppressed by a PAD inhibitor [26,27]. We also found that padi4 mRNA was significantly increased in pGIA joints and that most of the PAD4-expressing cells in joints were neutrophils [26,31]. Considering that PAD is essential to the process of NETosis, the source of the citrullinated protein in pGIA joints could be neutrophils.

Focusing on neutrophils in joints, we found that IL-6 receptor mRNA expression tended to be upregulated. Although we had previously confirmed that administration of IL-6 receptor antibodies suppressed arthritis in GPI-induced arthritis (GIA) [22], the effects in pGIA had not been investigated.

It has been reported that abatacept and rituximab can decrease ACPA titer [32,33]. This is mainly through a direct decrease in antibody production due to a decrease in B cells or plasmablasts, and given the mechanism of action of these biologics, they are unlikely to inhibit protein citrullination itself. In general, it is known that ACPA titer does not correlate with disease activity. It has also been reported that there are ACPAs that inhibit NETosis [34], called therapeutic ACPAs, suggesting that not all ACPAs may be involved in exacerbation of arthritis. Therefore, suppressing citrullinated protein production, rather than decreasing ACPA titer, is considered to be an appropriate therapeutic targets for RA.

IL-6 inhibition has proven to be very effective in RA [35], and its effects on neutrophils have been investigated, although to a lesser extent. Ruiz-Limón et al. reported that after 6 months of treatment with TCZ, a recombinant humanized anti-human IL-6 receptor monoclonal antibody, NETosis generation was reduced in RA patients and that treatment of RA neutrophils with IL-6 induced an increase in NET formation [36]. In addition, Yahagi et al. reported that IL-6 induced PAD4 expression in mouse neutrophils in vitro [29]. Thus, we hypothesized that IL-6 inhibition in pGIA results in the reduction in citrullinated protein via NET formation.

Indeed, inhibition of IL-6 signaling with anti-IL-6 receptor antibodies suppressed arthritis, with reduced neutrophil infiltration and NETosis. Since neutrophils in the joints tended to express higher levels of IL-6 receptors than neutrophils in the bone marrow in this study (Figure 2D), we speculate that there is some direct effect of IL-6 on neutrophils. One possible direct effect on neutrophils is the induction of NETosis. As we mentioned above, there have already been reports that IL-6 induces NETosis in human neutrophils [30,36] and that IL-6 induces PAD4 expression in mouse neutrophils [29], suggesting that IL-6 inhibition may directly inhibit NETosis. This scenario was supported in GIA-induced PAD4 knockout mice [37]. Arthritis, as well as IL-6 concentration in serum, was significantly reduced, and the survival of PAD4 knockout neutrophils was impaired [37]. Other indirect effects of IL-6 on neutrophils have been reported to include induction of IL-8 production and enhancement of ICAM-1 expression [38,39], thereby promoting neutrophil local infiltration. Based on these reports, we speculate that the decrease in the number of neutrophils in the joint and the direct inhibition of NETosis may have resulted in a decrease in citrullinated protein, a product of NETosis. However, further studies will be needed to elucidate the detailed mechanism.

Lastly, we confirmed the decrease in the 120KDa citrullinated protein in plasma (probably cit-ITIH4) in RA treated with TCZ. This clearly confirms that IL-6 inhibition suppressed plasma citrullinated protein which might be downregulated by NETosis in RA joints. Few reports have investigated fluctuation in ACPA titer before and after treatment in clinical practice, and they showed that ACPA titer did not change after anti-IL-6 medication [40,41]. As we mentioned above, ACPA titer does not correlate with disease activity and not all ACPAs may be involved in exacerbation of arthritis. On the other hand, the 120KDa citrullinated protein correlated with disease activity [26]. Further, we do not think that this protein necessarily represents the production of ACPAs, because this protein could be detected in RA patients who are negative for ACPAs. As a matter of fact, we found that the 120kDa citrullinated protein in the plasma of RA patients also decreased after treatment with a TNF inhibitor, infliximab, in our previous study [26]. Indeed, citrullinated proteins, including ITIH4, were mainly located in RA joints (not osteoarthritis joints) [31], and from our mouse analysis, this was mainly driven by synovium proliferation with neutrophil infiltration. A TNF inhibitor can regulate synovium proliferation, however, in this study, there was no upregulation of TNF receptor mRNA expression in articular neutrophils (Figure 2D). Therefore, we have hypothesized that IL-6 inhibitors might have a more direct effects on neutrophils.

## 4. Conclusions

In conclusion, we identified a specific increase in NETosis in pGIA joints, and suppression of arthritis by IL-6 blockade associated with decreased neutrophilic infiltration, NETosis in the joints, and plasma citrullinated protein. IL-6 signaling could have an important role in the production of citrullinated proteins via effects on neutrophil chemotaxis and their extracellular traps. Elucidation of the mechanism may provide a new insight into the regulation of neutrophil function in rheumatoid arthritis.

## 5. Materials and Methods

### 5.1. Mice

Male DBA/1 mice were purchased from Charles River Japan (Tokyo, Japan) and used at 6–10 weeks of age. All the mice were maintained under specific pathogen-free conditions at the University of Tsukuba.

Peptide GPI-induced arthritis was induced with interdermal injection of 25 μg of peptide GPI325–339 (pGPI) (Invitrogen, Carlsbad, CA, USA) in emulsified complete Freund’s adjuvant (CFA) (Becton Dickinson, San Jose, CA, USA). PBS was prepared as the vehicle control. Each mouse also received an injection of 200 ng of pertussis toxin (Sigma-Aldrich, St. Louis, MO, USA) intraperitoneally on days 0 and 2 after immunization to induce arthritis.

We evaluated arthritis visually and scored changes in each paw on a scale of 0 to 3 as follows: 0 = no evidence of inflammation, 1 = subtle redness or localized edema, 2 = easily identified swelling but localized to either the dorsal or the ventral paw surface, 3 = swelling of all aspects of the paws. We evaluated all 4 limbs, yielding a maximum possible score of 12 per mouse.

All the experimental protocols in this study were approved by the Institutional Animal Care and Use Committee of the University of Tsukuba (approval code: 20-188, approval date: 1 June 2020). All the animal experiments were conducted under institutional ethics guidelines.

### 5.2. Immunohistochemistry

The skin and ankle joints were fixed in neutralized 10% formalin, embedded in paraffin, and sliced. For immunohistological analysis using AMC antibodies, we prepared modification buffer by mixing Reagent A (20% H_2_SO_4_, 25% H_3_PO_4_, and 0.025%FeCl_3_) and Reagent B (1% diacetyl monoxime, 0.5% antipyrine, 1 M acetic acid) at a 2:1 ratio (volume/volume). The sections were covered with the modification buffer and incubated in a light-proof container at 37 °C for 2.5 h in order to modify citrulline residues. Then, the sections were incubated with rabbit AMC polyclonal antibodies diluted 1:3200 in 2% BSA in PBS to detect modified citrulline residues. Then, the sections were incubated with HRP-conjugated goat anti-rabbit IgG (H + L) (Bio-Rad, Hercules, CA, USA) for 30 min at room temperature. The sections were also stained with DAB (Nichirei Biosciences, Tokyo, Japan) and hematoxylin. To detect citrullinated histone 3 (CitH3), the sections were incubated with rabbit anti-CitH3 antibodies (Abcam, Cambridge, MA, USA) diluted 1:200 as primary antibodies. HRP-conjugated goat anti-rabbit immunoglobulin (IgG) (H + L) (Bio-Rad) diluted 1:1000 was used as a secondary antibody. The sections were also stained with DAB (Nichirei Biosciences) and hematoxylin.

### 5.3. Western Blot Analysis

The murine skin lysates were prepared by homogenization in PBS. The murine joint lysates were prepared by homogenization in RIPA buffer. Proteins were separated by SDS-PAGE, and then transferred to a PVDF membrane. For Western blot analysis of anti-CitH3 antibodies, 2.5 μg of proteins was loaded per well. The blots were then washed with 0.05% Tween-20 in TBS (TBST), blocked with 5% milk in TBST, and incubated overnight at 4 °C with anti-CitH3 antibodies diluted 1:1000 in 5% milk in TBST. After being washed, the blots were incubated with secondary antibodies consisting of HRP-conjugated goat anti-rabbit IgG (H + L) (Bio-Rad, Hercules, CA, USA) diluted 1:5000 in 5% milk in TBST for 1 h at room temperature. For Western blot analysis of sera using AMC antibodies, 100 μg of proteins was loaded per well. The modification buffer was added to the blots before incubation in a light-proof container at 37 °C for 2.5 h, in order to modify citrulline residues, as described above. Blots were washed with 0.05% TBST, and blocked with 5% milk in TBST, then incubated overnight at 4 °C with AMC antibodies diluted 1:3200 in 5% milk in TBST. After washing, the blots were incubated with secondary antibodies, HRP-conjugated goat anti-rabbit IgG (H + L) (Bio-Rad, Hercules, CA, USA) diluted 1:5000 in 5% milk in TBST, for 1 h at room temperature. Densitometric analysis was carried out using a FUSION FX7.EDGE (Vilber Lourmat, Marne-la-Vallée, France) with the band intensity determined by ImageJ software (National Institutes of Health, Bethesda, MD, USA).

### 5.4. Immunofluorescence Staining

To detect CitH3 and neutrophil elastase (NE), the paraffin-embedded sections of the murine joints were incubated for 90 min at room temperature with rabbit anti-CitH3 antibodies diluted 1:200 and with rat anti-NE antibodies (R&D Systems, Minneapolis, MN, USA) diluted 1:100 as primary antibodies. These samples were subsequently incubated with Alexa Fluor 546 anti-rabbit IgG (Invitrogen, Carlsbad, CA, USA) or Alexa Fluor 488 anti-rat IgG (Invitrogen) for 1 h at room temperature. The nuclei were counterstained with 4′,6-diamidino-2-phenylindole (DAPI). The stained slides were observed with an FV10i (Olympus, Tokyo, Japan).

### 5.5. Treatments with MR16-1

DBA/1 mice were immunized with pGPI as described above, and then treated with MR16-1 (an IgG1-specific mAb against murine IL-6R) or control IgG (Jackson ImmunoResearch, West Grove, PA, USA). The mice were injected intraperitoneally with 2 mg of MR16-1 or with control IgG on day 0. MR16-1 was a gift from Chugai Pharmaceutical (Tokyo, Japan)

### 5.6. Reverse Transcription Quantitative Polymerase Chain Reaction (PCR) Analysis

Cells were isolated from the bone marrow and joints of pGIA mice at day 14, and total RNA was then extracted from the cells using an RNeasy kit (Qiagen, Hilden, Germany) and transcribed into complementary DNA. Real-time quantitative polymerase chain reaction (RT-qPCR) was performed with QuantStudio 3 (Applied Biosystems, Foster City, CA, USA) using the TaqMan gene expression assay (Applied Biosystems).

### 5.7. Serum Samples

Serum samples were collected from 6 Japanese patients with RA, diagnosed by rheumatologists according to the 1987 American College of Rheumatology (ACR) classification criteria, or the 2010 ACR/EULAR classification criteria. This study was reviewed and approved by the ethics committee of the University of Tsukuba.

### 5.8. Statistical Analysis

All the data were expressed as means ± standard errors of the mean (SEMs). Differences between groups were evaluated for significance using the unpaired *t*-test, the ordinary one-way analysis of variance (ANOVA) with the post hoc Tukey test for multiple comparisons, and two-way ANOVA with the post hoc Sidak multiple comparisons test. *p* values less than 0.05 were considered to denote the presence of a significant difference. Statistical analyses were performed using Prism version 9 (GraphPad Software, San Diego, CA, USA).

## Data Availability

Data are contained within the article.
